# Genome-Wide Identification and Expression Profiles of Myosin Genes in the Pacific White Shrimp, *Litopenaeus vannamei*

**DOI:** 10.3389/fphys.2019.00610

**Published:** 2019-05-21

**Authors:** Xiaoxi Zhang, Jianbo Yuan, Xiaojun Zhang, Chengzhang Liu, Fuhua Li, Jianhai Xiang

**Affiliations:** ^1^Key Laboratory of Experimental Marine Biology, Institute of Oceanology, Chinese Academy of Sciences, Qingdao, China; ^2^Laboratory for Marine Biology and Biotechnology, Qingdao National Laboratory for Marine Science and Technology, Qingdao, China; ^3^University of Chinese Academy of Sciences, Beijing, China; ^4^Center for Ocean Mega-Science, Chinese Academy of Sciences, Qingdao, China

**Keywords:** myosin genes, muscle development, alternative splicing, penaeid shrimp, gene family

## Abstract

As the main structural protein of muscle fiber, myosin is essential for multiple cellular processes or functions, especially for muscle composition and development. Although the shrimp possess a well-developed muscular system, the knowledge about the myosin family in shrimp is far from understood. In this study, we performed comprehensive analysis on the myosin genes in the genome of the Pacific white shrimp, *Litopenaeus vannamei*. A total of 29 myosin genes were identified, which were classified into 14 subfamilies. Among them, *Myo2* subfamily was significantly expanded in the penaeid shrimp genome. Most of the Myo2 subfamily genes were primarily expressed in abdominal muscle, which suggested that Myo2 subfamily genes might be responsible for the well-developed muscular system of the penaeid shrimp. *In situ* hybridization detection showed that the slow-type muscle myosin gene was mainly localized in pleopod muscle and superficial ventral muscle of the shrimp. This study provides valuable insights into the evolutionary and functional characterization of myosin genes in shrimps, which provides clues for us to understand the well-developed muscular system of shrimp.

## Introduction

Nearly all eukaryotic cells possess myosin proteins, which bind to filamentous actin and produce physical forces through ATP hydrolysis (Richards and Cavalier-Smith, 2005; Sebé-Pedrós et al., 2014). Myosin plays key roles in muscle composition and various cellular activities, including cytokinesis, organelle transport, cell polarization, intracellular transport and signal transduction (Hofmann et al., 2009; Bloemink and Geeves, 2011; Hartman et al., 2011; Pette and Staron, 2015). The Myo2 subfamily (Myosin 2), also known as myosin heavy chain (MYH or MHC), are the main component of the contractile muscle. They are considered to be conventional myosins, while the other subfamilies are considered to be unconventional myosins. The Myo2 subfamily genes can be classified into three groups, including fast-type, slow-type and non-muscle type. The Myo2 subfamily proteins can form large bipolar filaments through tail-directed homo-oligomerization, while the tails of the unconventional myosins typically direct binding to membrane and other proteins (Woolner and Bement, 2009).

Myosin consists of three domains, including a conserved motor (or head) domain with actin-binding activities, a short neck that serves as a binding site for myosin light chains, and a variable tail that generally mediates interaction with the motor “cargo” to determine the functional specificity of the motor (Berg et al., 2001; Richards and Cavalier-Smith, 2005). In general, it is very difficult to investigate the myosin gene family systemically due to their extensive alternative splicing events, large sizes and high copy numbers. Although crustaceans are a large, diverse group, limited myosin genes have been reported, except that 17 myosin genes in 13 classes have been characterized in *Daphnia duplex* (Odronitz and Kollmar, 2008). In addition, a few *Myo2* sequences have been cloned from *Marsupenaeus japonicus*, *Penaeus monodon*, and *Litopenaeus vannamei*, respectively (Koyama et al., 2013). Hence, studies on the myosin gene family in crustaceans will enhance our understanding on their structure, function and evolution.

The Pacific white shrimp *L. vannamei* is one of the most economically important marine aquaculture species in the world (FAO, 2014). Based on the whole genome sequences (Zhang et al., 2019) and the transcriptome data, we performed a genome-wide analysis on the myosin gene family of *L. vannamei*. This study provides valuable resources for the shrimp myosin genes, which will increase our understanding on the crustacean muscular system.

## Materials and Methods

### Genome and Transcriptome Data

The genomic data of *L. vannamei* (PRJNA438564), *Lepeophtheirus salmonis* (GCA_001005205.1), *Parhyale hawaiensis* (GCA_001587735.1), *Eulimnadia texana* (GCA_002872375.1), were obtained from NCBI GenBank and Ensemble database. Our previous research conducted RNA-seq on several libraries, (I) five larval stages, including embryo, nauplius, zoea, mysis and post-larvae (Wei et al., 2014); (II) eight molting stages, including the inter-molt (C), pre-molt (D0, D1, D2, D3, D4), and post-molt (P1 and P2) stages (Gao et al., 2015); (III) 16 adult tissues, including antennal gland, brain, hemocyte, epidermis, eyestalk, gill, hepatopancreas, heart, intestine, abdominal muscle, lymphoid organ, ovary, stomach, testis, thoracic ganglion, and abdominal ganglion (Zhang et al., 2018).

### Isolation and Annotation of Myosin Genes

The full protein sequences of the *L. vannamei* genome were searched against the myosin head (motor domain) (PF00063) to find all the candidate myosin genes by using HMMER3.0 (Potter et al., 2018). All possible myosin transcripts were collected from the transcriptome data. Then, the redundant sequences were removed using CAP3 program (Huang and Madan, 1999). The candidate sequences were submitted to SMART (Letunic et al., 2014) and InterPro (Finn et al., 2016) databases to determine the integrity of the motor domain. More myosin sequences used in this study were collected from CyMoBase^1^ and (Hammesfahr et al., 2010).

### Gene Structure and Alternative Splicing

The characteristics of myosin genes, including the location, gene length, open reading frame (ORF), exon number and the number of deduced amino acids were analyzed in detail. To illustrate the structure of myosin genes, all alternative spliced exons were characterized by mapping all variant transcripts to the shrimp genome.

### Phylogenetic Analysis

The amino acid sequences of the conserved myosin head domain were aligned by MUSCLE program (version 3.8.31) (Edgar, 2004) with default parameters. A neighbor-joining (NJ) phylogenetic tree with 1000 bootstrap replicates was constructed by MEGA7.0 program (Kumar et al., 2016), and visualized using iTOL (Letunic and Bork, 2007). To investigate the evolution of the myosin gene family of shrimp, a class occurrence tree of 29 arthropods was generated by using the method described by Odronitz et al. (2009).

### Transcription Regulatory Element Identification

To investigate whether the expanded muscle-type Myo2 genes are associated with muscle composition or development, transcription regulatory elements of muscle-type Myo2 genes were predicted. In detail, the promoter regions, located at the 2 kb upstream of the transcriptional start site, were first extracted from the shrimp genome. The transcription regulatory elements were then predicted by Signal Scan^2^ with TRANSFAC database and the PATCH algorithm integrated in the GeneXplain platform^3^. In order to decrease the false positive rate, we intersected the predicted results of Signal Scan and GeneXplain.

### Expression Patterns at Early Development Stages and Different Tissues of Adults

In previous studies, we conducted Digital Gene Expression Profiling (DGE) to sequence 20 larval stages and 16 adult tissues of *L. vannamei*, and the RPKM (reads per kilobases per million reads) values of all transcripts were calculated (Zhang et al., 2018). The RPKM values of myosin genes were extracted and normalized with log2 conversion. Heat maps were created using TBtools software (Chen et al., 2018).

### *In situ* Hybridization

In this study, *LvMYH5* was characterized to be a slow-type muscle Myo2 gene. To further distinguish fiber types and muscle distribution of the abdominal muscle of shrimp, *in situ* hybridization was performed according to the protocol developed in our laboratory (Zhang et al., 2018). Briefly, primers LvMYH5-pF with T7 promoter sequence and LvMYH5-R (Supplementary Table 1) were designed to amplify the cDNA fragments as the template of *LvMYH5* to synthesize sense probe. Primers LvMYH5-pR with T7 promoter sequence and LvMYH5-F were designed as the template to synthesize antisense probe. PCR products were purified using MiniBEST DNA Fragment Purification Kit (Takara, Japan). The Digoxigenin (DIG)-labeled sense and antisense RNA probes were transcribed by TranscriptAid T7 High Yield Transcription Kit (Thermo Fisher Scientific, United States) and synthesized using DIG RNA Labeling Mixture (Roche, Germany), respectively, and stored at -80°C.

Transverse sections with 5–7 μm thickness were prepared from polyformaldehyde fixed abdominal muscle of juvenile shrimp (about 2 cm in body length) embedded in paraffin (Sigma, Germany), and hybridization was performed following the general protocol of DIG RNA labeling kit (SP6/T7) (Roche, Germany). The final concentration of both anti-sense and sense RNA probes were 1.5 ng/μl. Subsequently, alkaline phosphatase-conjugated anti-DIG antibody and NBT/BCIP (DIG Nucleic Acid Detection Kit, Roche) were used to detect RNA probes and observed under Nikon Eclipse 80i microscope (Nikon, Japan).

## Results

### Identification of Myosin Genes

**FIGURE 1:**
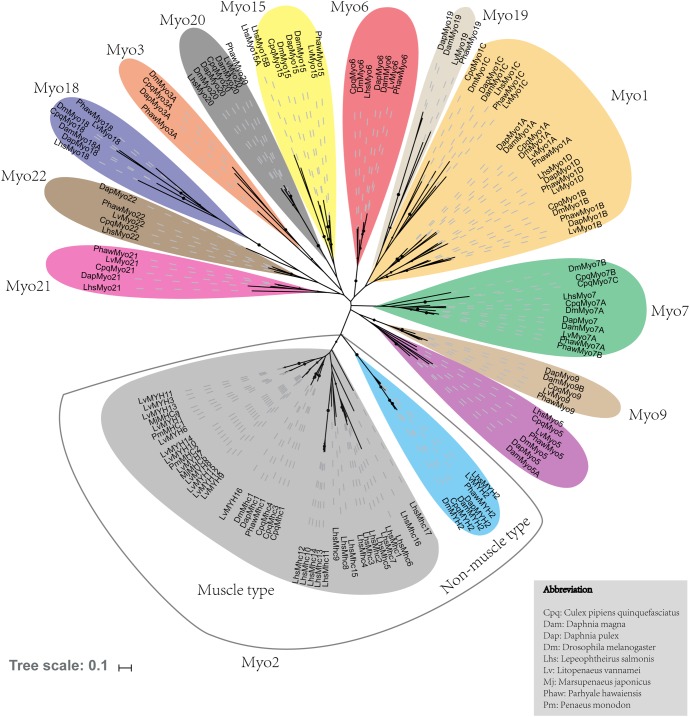
Phylogenetic tree of myosin head domains generated using the neighbor-joining method (MEGA7 software) with bootstrapping replication set at 1000. The myosin groups are identified in roman numerals beside each clade. Myosin genes from the following organisms were used: Cpq, *Culex pipiens quinquefasciatus*; Dam, *Daphnia magna*; Dm, *Drosophila melanogaster*; Dap, *Daphnia duplex*; Lhs, *Lepeophtheirus salmonis*; Lv, *Litopenaeus. vannamei*; Mj, *Marsupenaeus japonicus*; Phaw, *Parhyale hawaiensis*; Pm, *Penaeus monodon*.

A total of 29 myosin genes were identified in *L. vannamei*, including 15 muscle-type Myo2 genes, one non-muscle type Myo2 gene and 13 unconventional myosin genes (Table 1). Among them, five muscle-type myosin genes had been reported previously, including *LvMHC1*, *LvMHC2*, *LvMHC4, LvMHC5*, and *LvMHC6* (Koyama et al., 2012, 2013). They were designated as *LvMYH1*, *LvMYH7, LvMYH4, LvMYH5*, and *LvMYH6*, respectively, in this study to avoid confusion with major histocompatibility complex genes. The gene structure of these 29 myosin genes showed high complexity with the sizes ranging from 6.159 to 171.807 kb that corresponded to encode proteins of 1,010-2,158 aa (Table 1). Phylogenetic analysis indicated that these myosin genes were clustered into 14 distinct groups, namely 14 subfamilies, *Myo1, muscle-type Myo2, non-muscle type Myo2, Myo3, Myo5, Myo6, Myo7, Myo9, Myo15, Myo18, Myo19, Myo20, Myo21, and Myo22. Myo1* can be further divided into four subfamilies, *Myo1A*, *Myo1B*, *Myo1C*, and *Myo1D*. These myosin genes within each subfamily shared similar protein domains. Furthermore, *Myo1* and the muscle-type *Myo2* were the two largest clades including 22 and 40 myosin genes in the phylogenetic tree of this study, respectively. However, the smallest clade *Myo19* only included 4 myosin genes. All the 29 myosin genes identified in this study fell into 14 distinct clades and were closely related to the homologous proteins from other species (Figure 1). Of these genes, 15 muscle-type Myo2 genes were monophyletic with 27 muscle-type Myo2 genes from other arthropods.

**Table 1:** The 29 myosin genes identified from *L. vannamei* genome.

Myosin name	Genome position	Position (bp)	Exon	No. of aa	Unigenes
*LvMyo1A*	LVANscaffold_1022	139086: 159076	23	1081	Unigene0007554
*LvMyo1B*	LVANscaffold_2218	313258: 311201	20	1032	Unigene0049576
	LVANscaffold_3160	506207: 527443			
*LvMyo1C*	Unavailable	Unavailable	Unavailable	1177	c78174_g1
*LvMyo1D*	LVANscaffold_2215	1434634: 1447801	21	1177	c83379_g1
*LvMYH1*	LVANscaffold_3755	173364: 184470	22	1913	AB758443.1
*LvMYH2*	LVANscaffold_2946	891867: 984674	24	1972	c81769_g2
	LVANscaffold_675	114493: 156039			
*LvMYH3*	LVANscaffold_2985	675886: 686178	>18	1911	CL120.Contig45_All
*LvMYH4*	LVANscaffold_904	105970: 114423	11	1124	AB759099.1
*LvMYH5*	LVANscaffold_1838	9836: 15995	>8	>1198	AB759100.1
*LvMYH6*	LVANscaffold_2713	277521: 272168	22	1909	AB759104.1
		197752: 212409			
*LvMYH7*	LVANscaffold_2713	44648: 62854	22	1909	AB758444.1
*LvMYH8*	LVANscaffold_2713	169683: 186166	21	1909	Unigene7586_All
*LvMYH9*	LVANscaffold_904	190184: 192834	>21	1911	CL120.Contig24_All
		14479: 24317			
*LvMYH10*	LVANscaffold_904	26105: 36210	>14	1915	CL120.Contig4_All
*LvMYH11*	LVANscaffold_904	184800: 190271	19	1913	CL120.Contig83_All
		166283: 179232			
*LvMYH12*	LVANscaffold_904	236788: 225555	>20	1911	CL120.Contig50_All
*LvMYH13*	LVANscaffold_660	671473: 678521	>14	1911	CL120.Contig15_All
*LvMYH14*	LVANscaffold_1774	1247154: 1253260	>18	1929	CL120.Contig53_All
	LVANscaffold_854	418893: 427114			
*LvMYH15*	LVANscaffold_903	446522: 459841	>18	1909	CL120.Contig13_All
*LvMYH16*	LVANscaffold_1774	1258456: 1265373	>8	>1321	CL120.Contig78_All
*LvMyo5*	LVANscaffold_441	69978: 76891	37	1853	c81514_g1
		270006: 345054			
*LvMyo6*	LVANscaffold_1654	616383: 635879	29	1257	Unigene0049576
	LVANscaffold_1737	4719 : 14556			
*LvMyo7A*	LVANscaffold_2760	317572: 400940	40	2158	c83722_g3
*LvMyo9*	LVANscaffold_1465	684100: 748649	33	2057	c80593_g1
*LvMyo15*	LVANscaffold_2015	255392: 276113	20	1137	c83307_g1
*LvMyo18*	LVANscaffold_1503	325742: 153935	31	2037	c75923_g3
*LvMyo20*	LVANscaffold_609	944458: 956786	>18	1010	Unigene0091838
*LvMyo21*	LVANscaffold_2019	461705: 493440	26	1269	c83155_g1
*LvMyo22*	LVANscaffold_1625	630095: 647523	>35	1963	c83429_g1


**FIGURE 2:**
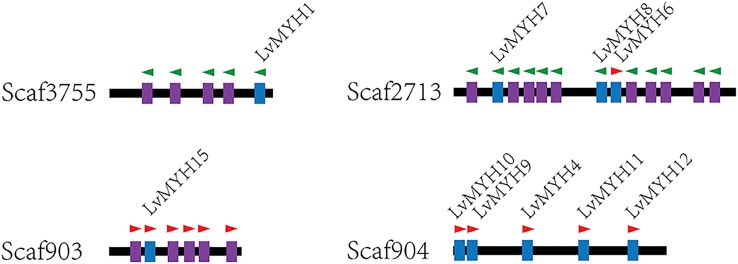
Representation of paralogous muscle-type Myo2 gene clusters arranged on different scaffolds of *L. vannamei*. Blue: the complete muscle-type MYH gene. Purple: fragmental Myo2-like gene sequences or pseudogenes of MYH. The green and red triangles represent transcription orientation.

Fifteen muscle-type Myo2 genes exhibited conspicuous lineage-specific expansion in the shrimp genome. Among them, eight Myo2 genes were tandemly distributed in two clusters (Figure 2), *LvMYH9*, *LvMYH10*, *LvMYH4*, *LvMYH11*, and *LvMYH12* were located on the Scaffold 904, *LvMYH7*, *LvMYH8*, and *LvMYH6* were located on the Scaffold 2713. Besides, at least 19 fragmental muscle-type *Myo2*-like sequences or pseudogenes were located on the Scaffold 903, Scaffold 2713 and Scaffold 3755 (Figure 2). The others scattered in the shrimp genome (Table 1).

### Myosin Functional Domain

**FIGURE 3:**
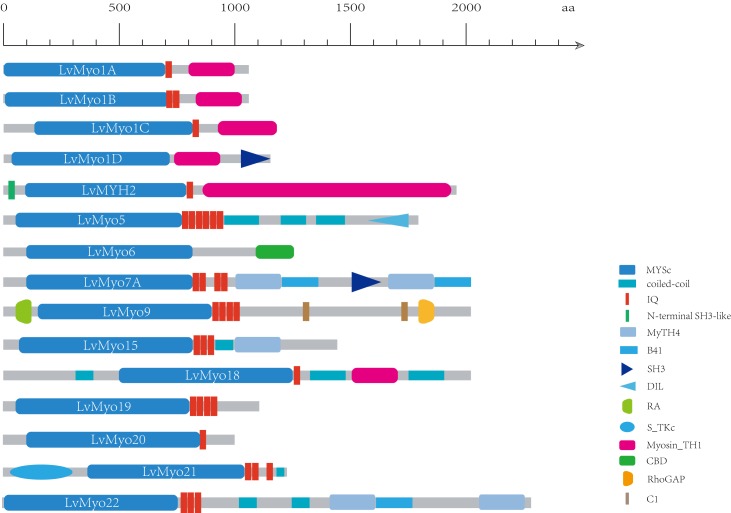
Schematic diagram of shrimp unconventional myosin structures. The putative domains or motifs were identified using the Pfam and SMART databases with the default parameters. MYSc, Myosin head, large ATPase; IQ, isoleucine-glutamine motif; DIL, dilute; CBD, cargo binding domain; MyTH4, myosin tail homology 4; B41, band 4.1, ezrin, radixin, and moesin; SH3, src homology 3; RA, Ras association (RalGDS/AF-6) domain; C1, Protein kinase C conserved region 1; RhoGAP, Rho GTPase-activating protein; S_TKC, Serine/Threonine protein kinases, catalytic domain. Myosin_TH1, myosin tail homology 1.

Generally, homologous myosin genes of different species possess similar domain architecture (Richards and Cavalier-Smith, 2005). All myosin genes identified in this study contained a large ATPase motor domain which hydrolyzes ATP, except for *LvMYH4*, which encodes a headless myosin. In addition, all the myosin genes contained at least one isoleucine-glutamine (IQ) motif except for *LvMyo6* (Figure 3). The myosin genes could be classified into different groups with diverse functions based on the remaining domains and variable number of IQ motifs. *LvMyo5* contained six IQ domains and a unique C-terminal DIL domain. *LvMyo19*, *LvMyo20*, and *LvMyo21* had truncated tail regions. Notably, all muscle-type Myo2 proteins contained an N-terminal SH3-like fold, a large ATPase motor domain, an IQ motif, and a large C-terminal myosin tail. However, the N-terminal SH3-like domain was absent in unconventional myosin genes of *L. vannamei*. Compared with *DamMyo18* and *DapMyo18*, the upstream PDZ domain was absent in *PhawMyo18* and *LvMyo18*.

### Phylogenetic Analysis

**FIGURE 4:**
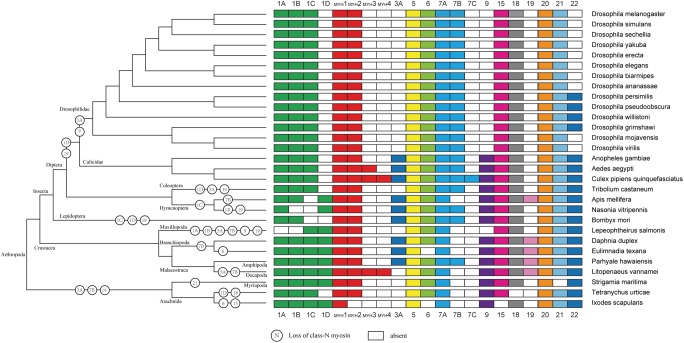
Myosin gain and loss in arthropod species. Tree on the left illustrates the phylogenetic relationships of species used (no scale). Colored boxes indicate the presence of certain myosin subfamily members in each species. Empty boxes mean the potential loss of particular myosin genes.

As expected, the crustacea formed a distinct clade from other arthropods, and *L. vannamei* was most closely related to *P. hawaiensis* belonging to Malacostraca clades (Figure 4). In addition, arthropods contained 14 classes (groups) of myosin genes including muscle-type *Myo2*, non-muscle *Myo2* (*MYH2*), *Myo5*, *Myo6, Myo7A, Myo15, Myo18*, *Myo20*, and *Myo21* with a few exceptions. For example, non-muscle type *Myo2*, *Myo6*, and *Myo15* were absent in *Ixodes scapularis*, and *Myo18* and *Myo21* were not found in *Tetranychus urticae* and *Strigamia maritima*, respectively. Most of these myosin genes were present in crustaceans, so we inferred that the last common crustacean ancestor had already contained these classes of myosin genes.

### Alternative Splicing

**FIGURE 5:**
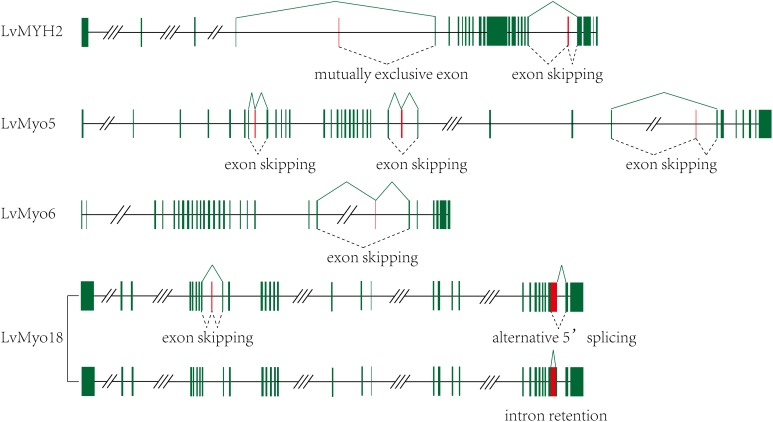
Transcript evidences and splicing patterns for unconventional myosin genes in *L. vannamei*. Green boxes: exons; red boxes: alternative splicing exons; line, introns. Three-slashes represent large intron, and two-slashes represent genomic gap.

Among the 29 myosin genes, four genes underwent alternative splicing (Figure 5 and Supplementary Table 2). The unspliced transcript of *LvMYH2* encoded 1,972 aa protein, and the corresponding spliced transcripts encoded 1,982 and 1,970 aa when the exon 20 was retained and exon 4 was mutually excluded, respectively. *LvMyo5* possessed at least three splicing events, the unspliced transcript encoded 1,853 aa and the corresponding spliced transcript encoded 1,833, 1,804 and 1,846 aa with the exon 7, 24 and 28 were skipped, respectively. In the case of *LvMyo6*, an exon skipping event occurred in exon 22, and the corresponding spliced transcript encoded 1,238 aa, whereas the unspliced transcript encoded 1,257 aa. As for *LvMyo18*, the unspliced transcript generated 2,037 aa in length. Exon 8 of *LvMyo18-A1* transcript was retained, and the corresponding spliced transcript encoded 2,061 aa. Alternatively, a novel 660 bp intron between exon 28 and exon 29 of *LvMyo18-A2* transcript was retained. Furthermore, an alternative acceptor site was found in exon 29 of *LvMyo18-A3* transcript, and the corresponding spliced transcript encoded 2,066 aa.

### Transcription Regulatory Elements of the Muscle-Type *Myo2* Promoters

**FIGURE 6:**
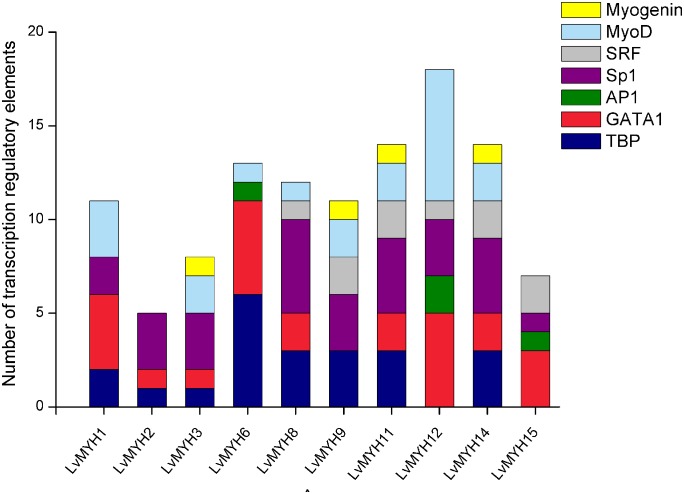
Transcription regulatory elements in the promoters of several muscle-type Myo2 genes that are related to muscle development. SRF, serum response factor; Sp1, specificity protein 1; AP1, activator protein 1; TBP, TATA-binding protein.

A large number of muscle development-related transcription factors binding to the upstream promoter sequences of 10 *LvMYH*s were identified, including TATA-binding protein (TBP), GATA-1, activator protein 1 (AP1), specificity protein 1 (Sp1), serum response factor (SRF), myogenic differentiation 1 (MyoD) and myogenin (Figure 6). *LvMYH1*, *LvMYH2*, *LvMYH3*, *LvMYH6*, *LvMYH8*, *LvMYH9*, *LvMYH11*, *LvMYH12*, *LvMYH14*, and *LvMYH15* possessed 11, 5, 8, 13, 12, 11, 14, 18, 14, and 7 binding sites of muscle-related transcription factors, respectively. Among them, *LvMYH12* contained the highest number of binding sites of five muscle-related transcription factors. In addition, all analyzed LvMYH genes possessed at least one binding site of MyoD, except for *LvMYH2* and *LvMYH15*. The *LvMYH3*, *LvMYH9*, *LvMYH11*, and *LvMYH14*, might be activated by myogenin (Figure 6).

### Temporal and Spatial Expression Patterns of Myosin Genes

**FIGURE 7:**
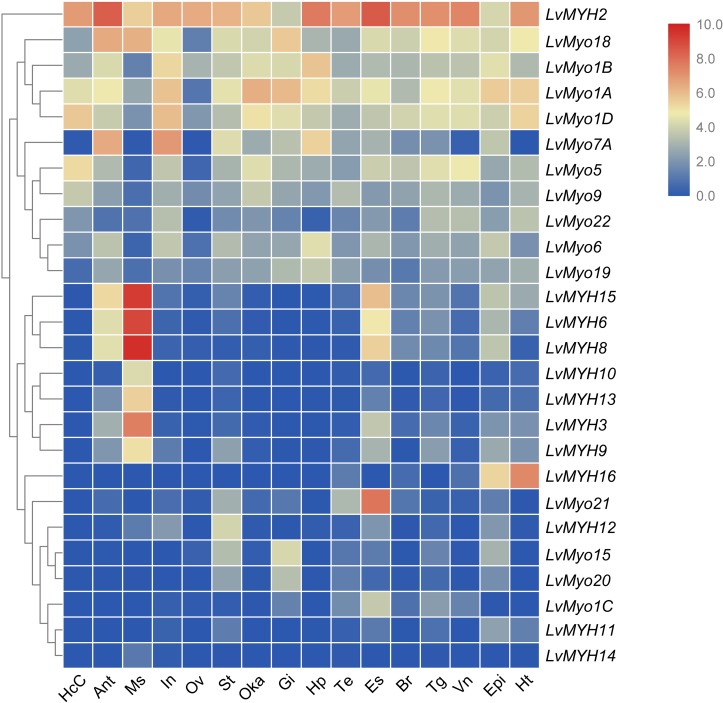
The expression profiles of myosin genes in 16 adult tissues. Log2-transformed expression values were used to create the heat map. The red or blue colors represent the higher or lower relative abundance of each myosin gene, respectively. To the left are myosin genes; the top lists the adult tissues of *L. vannamei*. (HcC, hemocyte cells; Ant, antennary gland; Ms, abdominal muscle; In, intestine; Ov, ovary; St, stomach; Oka, lymphoid organ; Gi, gill; Hp, hepatopancreas; Te, testis; Es, eyestalk; Br, brain; Tg, thoracic ganglion; Vn, ventral nerve; Epi, epidermis; Ht, heart).

We analyzed the temporal and spatial expression patterns of myosin genes based on the transcriptome of tissues or development stages (Figure 7). The results revealed that seven Myo2 subfamily genes, *LvMYH3*, *LvMYH6*, *LvMYH8*, *LvMYH9*, *LvMYH10*, *LvMYH13*, and *LvMYH15*, were specifically highly expressed in the abdominal muscle. *LvMYH12* was exclusively expressed in stomach, and *LvMYH16* was particularly abundant in heart and epidermis. The expression levels of *LvMYH11* and *LvMYH14* were very low in all tissues. Similarly, the unconventional myosin genes also showed tissue-specific expression patterns; for instance, *LvMyo5* was specifically expressed in hemocyte, and *LvMyo21* exhibited high expression level in eyestalk.

**FIGURE 8:**
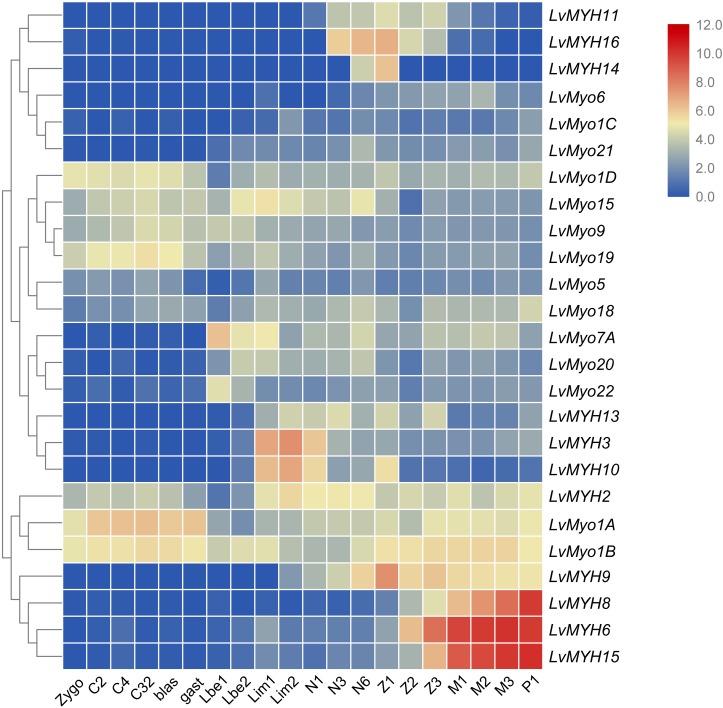
The expression profiles of myosin genes during development of *L. vannamei*. Log2-transformed expression values were used to create the heat map. The red or blue colors represent the higher or lower relative abundance of each myosin gene, repectively. On the right are myosin genes; the bottom lists different larval stages of *L. vannamei*, from left to right: zygote, 2 cells (C2), 4 cells (C4), 32 cells (C32), blastula (blas), gastrula (gast), limb bud embryo 1 (Lbe1), limb bud embryo 2 (Lbe2), larva in membrane 1 (Lim1), larva in membrane 2 (Lim2), Nauplius 1 (N1), Nauplius 2 (N2), Nauplius 3 (N3), Nauplius 6 (N6), Zoea 1 (Z1), Zoea 2 (Z2), Zoea 3 (Z3), Mysis 1 (M1), Mysis 2 (M2), Mysis 3 (M3), and Post-larva 1 (P1).

All myosin genes were detectable with dynamic expression patterns during the whole developmental stage. Most of muscle-type Myo2 genes were highly expressed between the larvae in membrane (Lim) stage and the post-larva stage (P1) (Figure 8). For instance, *LvMYH2*, *LvMYH3*, and *LvMYH10* were expressed from Lim stage to nauplius 6 (N6) stage, and *LvMYH6*, *LvMYH8*, *LvMYH9*, and *LvMYH15* were highly expressed from zoea 1 (Z1) to P1 stage. However, unconventional myosin genes exhibited a distinct pattern, *LvMyo1A*, *LvMyo1B*, *LvMyo1D*, *LvMyo9*, *LvMyo15*, and *LvMyo19* were mainly expressed from zygote to gastrula stage, and *LvMyo7A* and *LvMyo20* were expressed from limb bud embryo 1 (Lbe1) stage to N6 stage.

### *In situ* Hybridization

**FIGURE 9:**
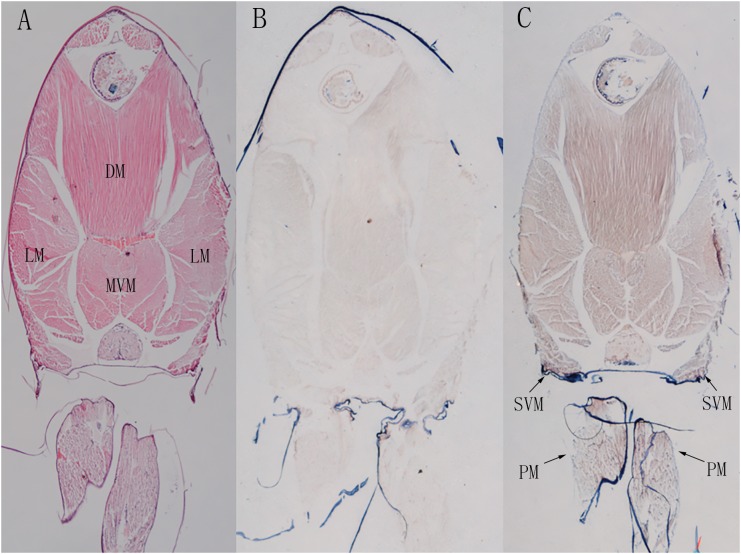
The localization of *LvMYH5* in the abdominal muscle and pleopod muscle of *L. vannamei*. **(A)** Hematoxylin-eosin (H&E) staining and **(B)** a sense probe were used as the control for **(C)** the antisense probe hybridization. LM, the lateral muscle; DM, the dorsal muscle; MVM, the main ventral muscle; PM, the pleopod muscle; SVM, the superficial ventral muscle. The picture of H&E staining was from our previous study (Zhang et al., 2018).

Shrimp abdominal muscle could be divided into four functional muscle groups, the superficial ventral muscle, the lateral muscle, the dorsal muscle and the main ventral muscle. The morphological organization of the skeletal muscle was displayed by the hematoxylin-eosin (H&E) staining (Figure 9A). *In situ* hybridization detection showed that *LvMYH5*, characterized as a marker gene of slow-type skeletal muscle Myo2, was mainly expressed in pleopod muscle and superficial ventral muscle (Figure 9C). They were attached between yokes of thin cuticle lying transversely across the posterior portion of each abdominal segment beneath the ventral nerve cord, and presumably function to hold the articular cuticle between the abdominal segments in place. However, no significant signal was observed in flexor and extensor muscle (Figure 9B).

## Discussion

### The Evolutionary Relationship of Myosin Genes in Arthropods

The evolutionary relationship of myosin family is limited in crustaceans due to the lack of Crustacea genome and difficulties for myosin gene identification. With the decoding of more and more Crustacea genomes, we are now in a position to validate the evolution of myosin genes proposed previously in arthropods and gain new insights in crustaceans. In accordance with previous study (Odronitz et al., 2009), all myosin subfamilies are shared in arthropods, suggesting that the common ancestor of arthropod has already owned all myosin classes (Odronitz and Kollmar, 2007). Meanwhile, the evolution of arthropods is likely accompanied by taxon- or species-specific losses of certain myosin classes. For example, Myriopoda and Arachnida appeared to have lost *Myo3A* and *Myo19* classes, and Drosophilidae have lost *Myo1D*, *Myo3A*, *Myo9*, and *Myo19* classes. As Crustacea, Maxillopoda may have lost *Myo1A* and *Myo1B* after separating from Branchiopoda and Malacostraca. The potential lack of the *Myo3* class in *L. vannamei* and *L. salmonis* may be specific characteristic of species. In agreement with the results of several comparative genome analyses (Hill, 2005; Colbourne et al., 2011; Chipman et al., 2014; Kao et al., 2016), the Crustacea clade is more closely related to the Hexapoda clade, but the Myriapoda clade is closer relative to the Arachnida.

### Gene Expansion of Muscle-Type *Myo2* Subfamilies

Gene family expansion generally results for a strengthened phenotype. In the *L. vannamei* genome, the muscle-type *Myo2* subfamilies showed significant expansion. Eight of 15 muscle-type Myo2 genes are tandemly duplicated. In addition, numerous fragmental muscle-type *Myo2*-like sequences or pseudogenes near the LvMYH cluster were also detected. Furthermore, most of the expanded muscle-type Myo2 genes possess binding sites of various myogenesis related transcription factors, such as Sp1 (Biesiada et al., 1999), AP-1 (Andreucci et al., 2002), myogenin (Hasty et al., 1993) and MyoD (Tapscott et al., 1988). It was reported that *LvMYH1* and *LvMYH7* were located in all fibers of abdominal muscles containing extensor and flexor muscles by *in situ* hybridization (Koyama et al., 2012, 2013). These results indicate that these expanded muscle-type Myo2 genes might be associated with muscle composition or development. In accordance with our previous study (Zhang et al., 2018), this study reveals the pleopod muscle and superficial ventral muscle of *L. vannamei* contain slow-type oxidative muscle fibers.

The muscle of shrimp accounts for more than half of the total body weight (Chen and Chen, 2001), but the mechanism of its well-developed muscular system is unknown. Our previous study has also indicated large expansion of actin gene family in *L. vannamei* (Zhang et al., 2018), which encodes structural proteins of muscle fiber together with muscle-type myosin heavy chains. Taken together, we speculate that the well-developed muscular system of *L. vannamei* might be associated with the massive expansion of muscle-type actin genes and muscle-type Myo2 genes.

### Alternative Splicing of Shrimp Myosin Genes

In most arthropods, MYH1 with multiple complex alternative splicing patterns was observed to be involved in muscle composition (Bernstein et al., 1986; Rozek and Davidson, 1986; Nyitray et al., 1994; Odronitz and Kollmar, 2008; Kollmar and Hatje, 2014). For example, the *D. duplex MYH1* (*Mhc1*) contains nine sets of mutually exclusive spliced exons (MXEs), and *Drosophila melanogaster MYH1* contains five clusters of MXEs and 480 combinations of alternative splicing patterns are possibly existed (Odronitz and Kollmar, 2008). Consistent with the report that crustacean *L. salmonis* contains 17 Myo2 genes without MXEs (Kollmar and Hatje, 2014), no MXE has been found in the 15 muscle-type Myo2 genes in this study. This implies that the last common ancestor of crustacean might have developed a MXE-less muscle-type Myo2 gene and followed by extensive gene duplications. Multiple, but not alternatively spliced myosin heavy chain genes are therefore a common characteristic of crustacean (Kollmar and Hatje, 2014). This might be caused by the loss of introns in the ancient muscle-type myosin heavy chain gene during arthropods evolution (Odronitz and Kollmar, 2008). However, unconventional myosin genes exhibited higher diversity due to the exon skipping in *L. vannamei*. It indicated that alternative splicing could potentially yield unconventional myosin variants with diverse functions.

### Special Expression Patterns of Shrimp Myosin Genes

Most of muscle-type Myo2 genes of *L. vannamei* were primarily expressed during larva in membrane and post-larvae stages. Combined with our previous study on actin gene family (Zhang et al., 2018), the whole early development stage of shrimp can be grouped into three periods. In the beginning of early development period (zygote to gastrula), most muscle-type Myo2 and actin genes are not expressed, then begin to be expressed in the second period (limb embryo and larvae in membrane stage), and their expression levels reach to the peak and remain at high levels in the third period (zoea to post-larvae stage). According to the report by Hertzler and Freas (2009), major ventral pleonal muscle and dorsal pleonal muscle of *L. vannamei* formed at the zoea stage, while pleopod muscle and major pleonal muscle formed in Mysis stage. Therefore, the high expression of muscle-type Myo2 and actin genes in these stages is compatible with the formation and development of pleonal and pleopod muscle.

The genes regulating muscle development, such as transcription factors, start to be expressed and to activate the co-expression of actin and myosin gene clusters at the nauplius stage. It promotes growth of muscle fibers in the larvae stage. Furthermore, compared with multiple alternative splicing of a single gene, the gene clusters have equivalent effects on improving the expression of transcripts, and strengthening the function of proteins (Talavera et al., 2007). Therefore, it is reasonable to conclude that the gene expansion of muscle-type Myo2 and actin genes may contribute to abundant fast-type muscle fibers and the explosive force of the shrimp abdominal muscles.

## Conclusion

In summary, we identified 29 myosin genes in the *L. vannamei* genome, and classified them into 14 subfamilies. Their genome localization, gene structure, protein domains, *trans-*acting elements in promoter regions and molecular evolution were comprehensively analyzed. Their temporal and spatial expression profiles provide insights into their important functions in muscle composition and development. Moreover, the expanded muscle-type Myo2 subfamily may explain the well-developed muscular system of *L. vannamei*. Collectively, this study will provide important clues for future research on the function of myosin genes in shrimp.

## Ethics Statement

This study was carried out in accordance with the recommendations of Welfare ethics of experimental animals and safety inspection system of animal experiments, laboratory animal management and ethics Committee of IOCAS. The protocol was approved by the laboratory animal management and ethics Committee of IOCAS.

## Author Contributions

JX, FL, and XnZ conceived and designed the study. XiZ and CL collected the data. XiZ conducted the bioinformatics analyses, performed all experiments and wrote the manuscript. JY, XnZ, and FL revised the manuscript.

## Conflict of Interest Statement

The authors declare that the research was conducted in the absence of any commercial or financial relationships that could be construed as a potential conflict of interest.

## Footnotes

1 https://www.cymobase.org/

2 https://www-bimas.cit.nih.gov/molbio/signal/

3 http://gene-regulation.com/
