# Supplementary material for: Genome-Wide Identification and Expression Profiles of Myosin Genes in the Pacific White Shrimp, Litopenaeus vannamei
Source: Front Physiol. 2019 May 21;10:610. doi: 10.3389/fphys.2019.00610 (PMC6537884; doi:10.3389/fphys.2019.00610)
Supplement: Supplementary file 1 [file Data_Sheet_1.docx]

**Supplementary Table 1.** Primers of *in situ* hybridization. T7 promoter sequences were underlined.

| Primer | Sequence (5’-3’) |
| --- | --- |
| LvMYH5-F | GCCCAGAACCAGGAGAAAATG |
| LvMYH5-R | TGACAGCAACCACACGGAAGA |
| LvMYH5-pF | TAATACGACTCACTATAGGGGCCCAGAACCAGGAGAAAATG |
| LvMYH5-pR | TAATACGACTCACTATAGGGTGACAGCAACCACACGGAAGA |

**Supplementary Table 2.** The corresponding transcripts of alternative spliced unconventional myosins genes.

| Alternative transcripts | Exon skipping | Intron retention | Alternative 5’ splicing | Mutually exclusive exon | No. of aa |
| --- | --- | --- | --- | --- | --- |
| LvMYH2-A1 | √ | — | — | — | 1962 |
| LvMYH2-A2 | — | — | — | √ | 1970 |
| LvMyo5-A1 | √ | — | — | — | 1833 |
| LvMyo5-A2 | √ | — | — | — | 1804 |
| LvMyo5-A3 | √ | — | — | — | 1846 |
| LvMyo6-A1 | √ | — | — | — | 1238 |
| LvMyo18-A1 | √ | — | — | — | 2061 |
| LvMyo18-A2 | — | √ | — | — | 2105 |
| LvMyo18-A3 | — | — | √ | — | 2066 |
